# Identification of an Actionable Mutation of KIT in a Case of Extraskeletal Myxoid Chondrosarcoma

**DOI:** 10.3390/ijms19071855

**Published:** 2018-06-23

**Authors:** Milena Urbini, Valentina Indio, Annalisa Astolfi, Giuseppe Tarantino, Salvatore Lorenzo Renne, Silvana Pilotti, Angelo Paolo Dei Tos, Roberta Maestro, Paola Collini, Margherita Nannini, Maristella Saponara, Ludovica Murrone, Gian Paolo Dagrada, Chiara Colombo, Alessandro Gronchi, Andrea Pession, Paolo Giovanni Casali, Silvia Stacchiotti, Maria Abbondanza Pantaleo

**Affiliations:** 1 “Giorgio Prodi” Cancer Research Center, University of Bologna, 40138 Bologna, Italy; valentina.indio2@unibo.it (V.I.); annalisa.astolfi@unibo.it (A.A.); giuseppe.tarantino6@unibo.it (G.T.); ludovica.murrone@studio.unibo.it (L.M.); andrea.pession@unibo.it (A.P.); 2 Department of Pathology, Fondazione IRCCS Istituto Nazionale dei Tumori, 20133 Milan, Italy; salvatore.renne@humanitas.it (S.L.R.); silvana.pilotti@istitutotumori.mi.it (S.P.); paola.collini@istitutotumori.mi.it (P.C.); gianpaolo.dagrada@istitutotumori.mi.it (G.P.D.); 3 Department of Pathology, Treviso General Hospital, 31100 Treviso, Italy; angelopaolo.deitos@aulss2.veneto.it; 4 Unit of Experimental Oncology 1, CRO Aviano National Cancer Institute, 33081 Aviano, Italy; rmaestro@cro.it; 5 Department of Specialized, Experimental and Diagnostic Medicine, Sant’Orsola-Malpighi Hospital, University of Bologna, 40138 Bologna, Italy; margherita.nannini@unibo.it (M.N.); maristella.saponara@unibo.it (M.S.); maria.pantaleo@unibo.it (M.A.P.); 6 Department of Surgery, Fondazione IRCCS Istituto Nazionale dei Tumori, 20133Milan, Italy; chiara.colombo@istitutotumori.mi.it (C.C.); alessandro.gronchi@istitutotumori.mi.it (A.G.); 7 Department of Cancer Medicine, Fondazione IRCCS Istituto Nazionale dei Tumori, 20133Milan, Italy; paolo.casali@istitutotumori.mi.it (P.G.C.); silvia.stacchiotti@istitutotumori.mi.it (S.S.)

**Keywords:** *KIT*, EMC, extraskeletal myxoid chondrosarcoma, next generation sequencing

## Abstract

Extraskeletal myxoid chondrosarcoma (EMC) is an extremely rare soft tissue sarcoma, marked by a translocation involving the *NR4A3* gene. EMC is usually indolent and moderately sensitive to anthracycline-based chemotherapy. Recently, we reported on the therapeutic activity of sunitinib in a series of EMC cases, however the molecular target of sunitinib in EMC is unknown. Moreover, there is still the need to identify alternative therapeutic strategies. To better characterize this disease, we performed whole transcriptome sequencing in five EMC cases. Peculiarly, in one sample, an in-frame deletion (c.1735_1737delGAT p.D579del) was identified in exon 11 of *KIT*. The deletion was somatic and heterozygous and was validated both at DNA and mRNA level. This sample showed a marked high expression of *KIT* at the mRNA level and a mild phosphorylation of the receptor. Sanger sequencing of *KIT* in additional 15 Formalin Fixed Paraffin Embedded (FFPE) EMC did not show any other mutated cases. In conclusion, exon 11 *KIT* mutation was detected only in one out of 20 EMC cases analyzed, indicating that *KIT* alteration is not a recurrent event in these tumors and cannot explain the EMC sensitivity to sunitinib, although it is an actionable mutation in the individual case in which it has been identified.

## 1. Introduction

Extraskeletal myxoid chondrosarcoma (EMC) is an extremely rare soft tissue sarcoma, marked by a translocation involving the *NR4A3* gene [1,2]. In most cases (about 62–75%), *NR4A3* fuses with *EWSR1* on chromosome 22 [3], and less frequently (27%) with *TAF15* on chromosome 17. More rarely, other fusion partners of *NR4A3* were identified, namely *TCF12*, *TFG*, and *HSPA8* [3,4,5,6,7].

Despite the high risk of metastases (about 40%), EMC are usually indolent with 10-year survival ranging between 65% and 85% [8,9]. Generally, these sarcomas are moderately sensitive to anthracycline-based chemotherapy [10], and therefore there is an urgent need to identify alternative therapeutic strategies [8,9,10]. Recently, we reported the therapeutic activity of sunitinib in a cohort of 10 EMC patients with 6 partial responses, 2 stable disease, and 2 showed progression. In that series, the two progressive patients upon treatment were those carrying a *TAF15*-*NR4A3* fusion, while all the others were *EWSR1*-positive [11,12]. EMC tumor specimens showed RET proto-oncogene expression and activation, while no other predictive biological markers of response were identified [12,13]. To further investigate the molecular alterations present in *EWSR1*-positive EMC cases, we performed whole transcriptome sequencing (WTS) in a small series of EMC, identifying a case carrying an activating *KIT* mutation and the recurrence of this event was evaluated in a larger series.

## 2. Results and Discussion

**Figure 1:**
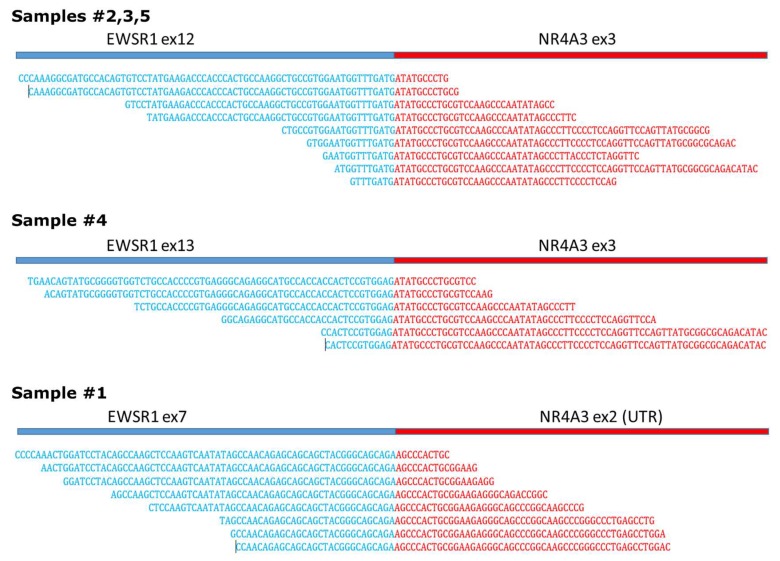
Three different *EWSR1-NR4A3* breakpoints identified through whole transcriptome sequencing (WTS).

WTS was performed on five cases of EMC positive for *EWSR1*-*NR4A3* fusion. Presence of the chimeric mRNA was confirmed and no additional fusion event was identified. In concordance with previous findings, as reported in the COSMIC database (available online: cancer.sanger.ac.uk/cosmic), exon12/exon3 *EWSR1*-*NR4A3* fusion was the most frequent breakpoint in our cohort. In particular, the exon12/exon3 junction was detected in three out of five cases (#2, #3, #5) while exon13/exon3 and exon7/exon2 were detected respectively in samples #4 and #1 (Figure 1). All fusions retained the coding frame for creating a chimeric protein, with the exception of sample #1, in which *EWSR1* fuses with exon2 of *NR4A3*, an exon upstream of the start codon.

**Figure 2:**
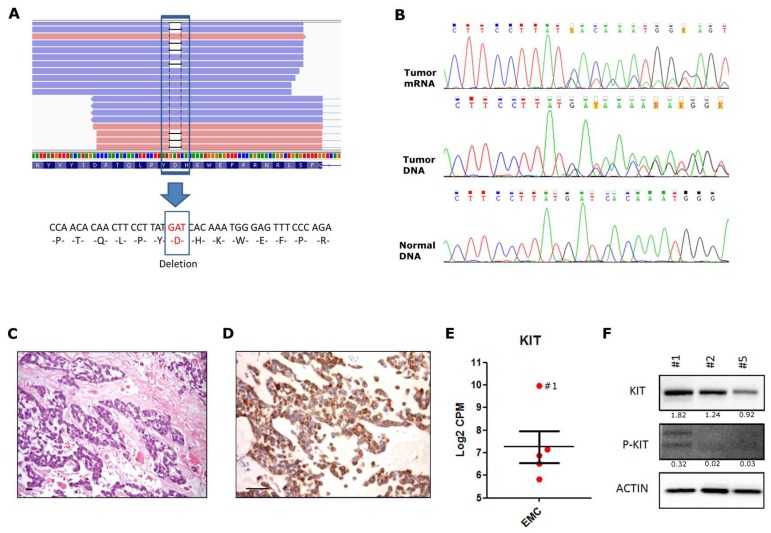
Identification of a KIT exon 11 deletion in one case of EMC. (**A**) WTS reads supporting the deletion. Blue and pink bars represented the reads (respectively sense and antisense to the reference). Black lines that interrupt the alignment of the reads represent the deletion of three bases, corresponding to p.D579del. (**B**) Sanger sequencing validation of the mutation at mRNA and DNA level (upper and lower panel). In tumor specimens, the overlapping of two signals, starting after the TAT codon, demonstrated the presence of the heterozygous deletion corresponding to p.D579del. (**C**) Hematoxylin and eosin stain, and (**D**) immunohistochemistry evaluation of *KIT* in Sample #1. Scale-bars indicate 50 μm (**E**) Expression level of *KIT* mRNA was evaluated through counts per million (CPM) analysis from WTS data. Each red circle correspond to one of the five EMC case analyzed: sample #1 is indicated. (**F**) Detection of KIT protein expression and phosphorylation through western blot on three EMC cases. Relative quantification of band intensity is shown under each blot. Actin was used as the loading control.

Pathogenic single nucleotide variants (SNV) and insertions and deletions (INDEL) were searched starting from WTS data. Interestingly, in one case an in-frame deletion (c.1735_1737delGAT p.D579del) was detected in the juxtamembrane domain (exon 11) of the KIT receptor (Figure 2A). The mutation was somatic and heterozygous, and was validated both at the DNA and mRNA level through Sanger sequencing (Figure 2B). This EMC case showed epithelioid morphology with cells arranged in cords and strands (Figure 2C), with diffuse cytoplasmic and membranous immunohistochemical positivity for KIT (Figure 2D). We evaluated the *KIT* expression using WTS data, finding that sample #1 had an extremely high mRNA level, about 9.8-fold greater than in the other EMC cases (Figure 2E). At the protein level, KIT was found to be expressed in all three EMC samples tested, while a mild phosphorylation of KIT was detected only in the KIT mutated case (Figure 2F).

*KIT* encodes a receptor tyrosine kinase, which is often mutated in gastrointestinal stromal tumors (GIST) and represents the target and rationale of tyrosine kinase inhibitors (TKI) treatment in this tumor. While 85% of GISTs are characterized by alteration of *KIT*, more rarely (5–10% of cases) they can harbor activating mutations of platelet derived growth factor receptor alpha (*PDGFRA*) [14,15]. 

With the aim to evaluate the frequency of the involvement of *KIT* or *PDGFRA* alterations in EMC, an additional cohort of 15 EMC were collected. Through Sanger sequencing, we analyzed all the known hotspot exons of *KIT* and *PDGFRA* throughout the entire cohort, however we failed to detect any other mutated case in addition to sample #1. These data supported the hypothesis that alterations of *KIT/PDGFRA* are extremely rare in EMC.

In GIST, the location of the *KIT* mutation is clinically relevant as it influences the response to imatinib. Mutations of *KIT* exon 11 are the most frequent GIST (about 70%), and generally they are the most sensitive to imatinib [15]. The p.D579del, described in this EMC case, is a known gain-of-function mutation of KIT through disruption of the autoinhibitory function of the juxtamembrane domain [16], and it was already reported in several cases of GIST and of melanoma (COSM1294). Moreover, it was also described in a metastatic case of sinonasal carcinoma and in a heavily pretreated thymic carcinoma. Noticeably, also in these two types of tumors, authors observed a relevant disease stabilization under imatinib [17,18]. The EMC patient with a *KIT* exon 11 mutation described here never received imatinib. Thus, the therapeutic role of this agent in this case is still to be defined. Interestingly the same patient had been treated with sunitinib with a prolonged response. Unfortunately, the *KIT* exon 11 mutation was found only in one case out of 20, and therefore cannot explain the other high frequency of responses to sunitinib that have been observed, although it cannot be ruled out that it may have favored the response in this single case.

## 3. Materials and Methods

### 3.1. Samples

Five *EWSR1*-*NR4A3* positive EMCs were collected for WTS analysis. As validation analysis, we evaluated the frequency of a *KIT* alteration in EMC in an additional retrospective cohort of 15 FFPE cases. The study was approved by the local ethics committee, and informed written consent was obtained in all cases, in accordance with national legislation and the Helsinki Declaration (27 May 2014, Prot. INT 54/13). Sample characteristics are shown in Supplementary Materials Table S1.

### 3.2. Whole Transcriptome Sequencing

For WTS analysis, total RNA was extracted from tumor specimens with RNeasy Mini Kit (Qiagen, Milan, Italy), then cDNA libraries were synthesized from 250 ng of total RNA with TruSeq RNA Sample Prep Kit v2 (Illumina, San Diego, CA, USA) according to the manufacturer’s recommendations. Briefly, poly(A)-RNA molecules were purified using oligo-dT magnetic beads, then mRNA was fragmented and randomly primed for reverse transcription, followed by second-strand synthesis to create double-stranded cDNA fragments. The generated cDNA fragments went through a terminal-end repair process and ligation using paired-end sequencing adapters, then amplified to create the final cDNA library. WTS libraries were quality-checked and sized with Agilent DNA 7500 chips on the Bioanalyzer 2100 (Agilent Technologies, Milan, Italy), then quantified using a fluorometric assay (QuantITPicogreen assay, Thermo Fisher, Monza, Italy). Then, 12 pM paired-end libraries were amplified and ligated to the flowcell by bridge PCR, and sequenced at 2 × 80 bp read length for WTS, using Illumina Sequencing by synthesis (SBS) technology.

### 3.3. Bioinformatic Analysis

After demultiplexing and FASTQ generation, the paired-end reads were trimmed using AdapterRemoval (available online: https://github.com/MikkelSchubert/adapterremoval). Sequences were mapped to HG38 with the TopHat/BowTie (available online: https://ccb.jhu.edu/software/tophat/) and the PCR and optical duplicates were removed with the function rmdup of Samtools (available online: https://samtools.sourceforge.net). For fusion genes detection, DeFuse (available online: https://sourceforge.net/projects/defuse), ChimeraScan (available online: http://chimerascan.googlecode.com), Tophatfusion (available online: https://ccb.jhu.edu/software/tophat/fusion_index.html), and FusionMap (available online: http://www.omicsoft.com/fusionmap) were used. Variation calling was performed with SAMtools and SNVMix2, thus identifying all the point mutations, insertions, and deletions present in the sample (SNV and InDels). Variants present in dbSNP, Exac, and Exome Variant Server (EVS) with frequency greater than 1% were excluded. For evaluation of *KIT* gene expression level, htseq-count function (Python package Htseq—available online: http://www.huber.embl.de/HTSeq/doc/overview.html) was used.

### 3.4. Sanger Sequencing

*KIT* and *PDGFRA* hotspot exons were sequenced through Sanger method in these cases and in an additional cohort of 15 EMC FFPE samples. DNA was extracted with a QiAmp DNA Mini Kit (Qiagen, Milan, Italy) from fresh frozen tissue and with QiAmp DNA Micro Kit (Qiagen)) for FFPE. Respectively, 10 and 20 ng of DNA was used for PCR using primers specific for exons 8, 9, 11, 13, 14, and 17 of *KIT*, and for exons 12, 14, and 18 of *PDGFRA* (Supplementary Materials Table S2). Amplicons were then purified and sequenced on a ABI3730 Genetic Analyzer (Applied Biosystems, Monza, Italy).

### 3.5. Immunohistochemistry

KIT protein expression was assessed by immunohistochemistry on FFPE 3 µm slides using c-kit (Ab CD117, A4502, Agilent Technologies Dako, Milan, Italy) with an automated immunostainer (BenchMark Ultra, Ventana Medical Systems Inc., Tucson, AZ, USA) according to manufacturer’s instructions.

### 3.6. Western Blot

Frozen tumor samples were homogenized in a RIPA buffer containing phosphatase and protease inhibitors (Halt™ Protease and Phosphatase Inhibitor Cocktail, ThermoFisher) and immunoblotted. ImageJ (available online: https://imagej.nih.gov/ij/) was used for signal quantification and relative quantification was calculated in comparison with β-Actin. The following primary antibodies were used: CD117 (A4502, Agilent Technologies Dako), Phospho-c-KIT (#3391, Cell Signaling, Leiden, The Netherlands), β-Actin (A1978, Sigma-Aldrich, Milan, Italy).

## 4. Conclusions

In conclusion, this is the first report of the presence of *KIT* exon 11 mutations in EMC. This event was detected in one out of 20 EMC cases analyzed, indicating that a *KIT* alteration is not a recurrent event in EMC, and cannot explain the EMC sensitivity to sunitinib, although it is an actionable mutation in the individual case in which it has been identified.

## Supplementary Materials

Supplementary materials can be found at http://www.mdpi.com/1422-0067/19/7/1855/s1.
