# Supplementary material for: Identification of an Actionable Mutation of KIT in a Case of Extraskeletal Myxoid Chondrosarcoma
Source: Int J Mol Sci. 2018 Jun 23;19(7):1855. doi: 10.3390/ijms19071855 (PMC6073125; doi:10.3390/ijms19071855)
Supplement: Supplementary file 1 [file ijms-19-01855-s001.pdf]

International Journal of *Molecular Sciences*

MDPI

Supplementary Materials: Identification of An Actionable Mutation of KIT in a Case of Extraskeletal Myxoid Chondrosarcoma

Table S1. Clinical characteristics.

Sample	EMC Diagnosis with NR4A3 Fusion Y/N	SEX	Age at Time of Primary Tumor	Primary Tumor Site	Distant Relapse Y/N (Site)
#1	Y	F	48	thigh	Y (abdomen)
#2	Y	M	55	thigh	Y (LN)
#3	Y	M	49	groin	Y (lung)
#4	Y	M	52	leg	N
#5	Y	M	56	thigh	Y (lung)
#6 *	Y	M	59	thigh	Y (lung)
#7 *	Y	M	48	thigh	N
#8 *	Y	M	35	thoracic wall	Y (lung)
#9 *	Y	M	72	thigh	Y (lung, LN)
#10 *	Y	M	77	leg	Y (lung)
#11 *	Y	F	64	thigh	N
#12 *	Y	M	73	thigh	Y (lung)
#13 *	Y	M	73	leg	N
#14 *	Y	F	39	leg	N
#15 *	Y	M	60	leg	Y (lung)
#16 *	Y	M	41	groin	N
#17 *	Y	M	71	thigh	Y (lung)
#18 *	Y	M	41	groin	N
#19 *	Y	F	73	leg	UKN
#20 *	Y	M	44	lumbar region	UKN

* Sample analyzed only by Sanger sequencing of *KIT* and *PDGFRA* exons hot spot of mutation.

Table S2. Primers used for Sanger sequencing of KIT and PDGFRA exons hot spot of mutation.

Target	Forward	Reverse
KIT		
Exon 8	ACTTGCTCCCTCAGGCTACTCAG	CAGTCCTTCCCCTCTGCATTATA
Exon 9	CCTAGAGTAAGCCAGGGCTTTTG	CAGAGCCTAAACATCCCCTTAAAT
Exon 11	TCTCTCTCCAGAGTGCTCTAATGAC	AAGGAAGCCACTGGAGTTCC
Exon 13	CGCTTGACATCAGTTTGCCA	AGAGAGAACAACAGTCTGGGT
Exon 14	TAATGGCCATGACCACCCTT	CCCATGAACTGCCTGTCAAC
Exon 17	TGGTTTTCTTTTCTCCTCCAACC	TGCAGGACTGTCAAGCAGAGA
PDGFRA		
Exon 12	TGTGGAGTGAACGTTGTTGG	GTGTGCAAGGGAAAAGGGAG
Exon 14	CAGGAAGTTGGTAGCTCAGC	CAACCACATGTGTCCAGTGA
Exon 18	CCAGTCTTGCAGGGGTGAT	GGCACCGAATCTCTAGAAGC
